# PRRSV Promotes MARC-145 Cells Entry Into S Phase of the Cell Cycle to Facilitate Viral Replication *via* Degradation of p21 by nsp11

**DOI:** 10.3389/fvets.2021.642095

**Published:** 2021-03-24

**Authors:** Xuexia Wen, Xinna Ge, Lei Zhou, Yongning Zhang, Xin Guo, Hanchun Yang

**Affiliations:** ^1^Key Laboratory of Animal Epidemiology of the Ministry of Agriculture and Rural Affairs, College of Veterinary Medicine, China Agricultural University, Beijing, China; ^2^Key Laboratory of Livestock Infectious Diseases in Northeast China, Ministry of Education, College of Animal Science and Veterinary Medicine, Shenyang Agricultural University, Shenyang, China

**Keywords:** PRRSV, cell cycle, replication, non-structural protein 11, proteasomal degradation

## Abstract

Porcine reproductive and respiratory syndrome virus (PRRSV) remains one of the most economically significant pathogens that seriously affect the global swine industry. Despite sustained efforts, the factors that affect PRRSV replication in host cells are far from being fully elucidated and thus warrants further investigation. In this study, we first demonstrated that PRRSV infection can cause downregulation of endogenous p21 protein in MARC-145 cells in a virus dose-dependent manner. Next, we analyzed the effect of p21 knockdown by RNA interference on cell cycle progression using flow cytometric analysis, and found that knockdown of p21 promotes MARC-145 cells entry into S phase of the cell cycle. Interestingly, we further discovered PRRSV infection is also able to promote MARC-145 cells entry into the S phase. Subsequently, we synchronized MARC-145 cells into G0/G1, S and G2/M phases, respectively, and then determined PRRSV replication in these cells. Results here show that the MARC-145 cells synchronized into the S phase exhibited the highest viral titer among the cells synchronized to different phases. Additionally, to reliably analyze the potential role of endogenous p21 protein in PRRSV replication, we constructed a p21 gene-knockout MARC-145 cell line (p21^−/−^) using CRISPR/Cas9 technology and evaluated its capability to support PRRSV replication. Our results indicate that knockout of p21 is conducive to PRRSV replication in MARC-145 cells. Furthermore, through construction of a series of eukaryotic plasmids expressing each of individual PRRSV proteins combined with cell transfection, we demonstrated that the nonstructural protein 11 (nsp11) of PRRSV mediates p21 degradation, which was further confirmed by generating a stable MARC-145 cell line constitutively expressing nsp11 using a lentivirus system. Notably, we further demonstrated that the endoribonuclease activity rather than the deubiquitinating activity of nsp11 is essential for p21 degradation via mutagenic analysis. Finally, we demonstrated that nsp11 mediates p21 degradation via a ubiquitin-independent proteasomal degradation manner. Altogether, our study not only uncovers a new pathogenesis of PRRSV, but also provides new insights into development of novel antiviral strategies.

## Introduction

Porcine reproductive and respiratory syndrome (PRRS) has been one of the most economically important diseases affecting the global swine industry for three decades (1, 2). The typical clinical signs of PRRS include reproductive failures in sows and respiratory disorders in pigs of all ages (2). The causative agent of PRRS, porcine reproductive and respiratory syndrome virus (PRRSV), is classified in the family *Arteriviridae* in the order *Nidovirales* (3). Recently, the existing two different species of PRRSV, formerly named PRRSV-1 and PRRSV-2, were taxonomically classified as *Betarterivirus suid 1* and *Betarterivirus suid 2*, respectively, in the genus *Betaarterivirus* (https://talk.ictvonline.org/ICTV/proposals/2017). The virus contains a linear, non-segmented, single-stranded, positive-sense RNA genome of approximately 15 kb, which consists of 11 identified open reading frames (ORFs) (1), among which ORF1a and ORF1b occupy the 5′-proximal ~75% of viral genome and encode two large nonstructural polyproteins—pp1a and pp1ab. The two polyproteins are then processed by viral encoded proteases to produce at least 16 distinct non-structural proteins (nsps), including nsp1α, nsp1β, nsp2, nsp2N, nsp2TF, nsp3, nsp4, nsp5, nsp6, nsp7α, nsp7β, nsp8, nsp9, nsp10, nsp11, and nsp12 (4–13). These nsps have been shown to be involved in replication and transcription of viral genome (4, 6), in modulation of host immunity (1, 4), and in viral pathogenesis and virulence (1, 14, 15). The remaining ORFs encode eight viral structural proteins, including five minor envelope proteins (GP2a, GP3, GP4, E, and ORF5a), two major envelope proteins (GP5 and M), and the nucleocapsid protein (N) (1, 16).

As an important infectious disease that has caused colossal economic losses to the global pig production (17, 18), although great efforts have been made to explore the pathogenesis of PRRSV from diverse aspects (2), the factors that affect the replication and pathogenicity of PRRSV are far from being fully elucidated and thus warrants further investigation. To date, several cellular response mechanisms, such as apoptosis and autophagy, have been demonstrated to be hijacked by PRRSV to benefit its own replication (19–22). Moreover, PRRSV infection was shown to be able to inhibit MARC-145 proliferation by inducing cell cycle arrest at G2/M phase via activation of the Chk/Cdc25C and p53/p21 pathway, which is thus beneficial to PRRSV replication (23). In addition, a cellular transcriptional analysis of nsp11-expressing MARC-145 cells using RNA microarrays showed that the PRRSV nsp11 protein can cause delay of cell cycle progression at the S phase (24), however, the biological significance of this phenomenon remains unknown.

Cell cycle is a complex physiological process through which a cell duplicates its genome, grows, and divides into two daughter cells (25). The mammalian cell cycle consists mainly of four distinct phases: gap 1 (G1), synthesis (S), gap 2 (G2), and mitosis (M) (25, 26). Most cells are normally maintained in a quiescent state, which is also referred to as the G0 phase. When being stimulated by intracellular or extracellular stimuli, the quiescent cells exit the G0 phase and enter the cell cycle (25). The transition from one cell cycle phase to the next is a highly ordered and tightly regulated process, which involves various functional complexes formed by several cyclin-dependent kinases (CDKs) and their regulatory partner proteins, the cyclins (27, 28). Although the levels of CDKs maintain constant throughout the cell cycle, the activity of CDKs periodically oscillate caused by cell cycle-dependent phosphorylation and changes in the amounts of cyclins (25). Upon stimulation with mitotic signals, the cells in the early G1 phase begin to synthesize D-type cyclins, which are assembled with CDK4/6 to form an active CDK complex that proceeds to phosphorylate the retinoblastoma protein (pRb). Phosphorylation inactivates pRb and abrogates its interaction with E2F complex, leading to the release of pRb-bound E2F transcription factors. The dissociated E2F then initiates the transcription of the next wave of cyclins including cyclins E1 and E2, and other genes required for the G1-to-S-phase transition (29). The cyclin E-CDK2 complex has been shown to play a crucial role in regulating the cell cycle from G1 to S phase (30). In addition to cyclins, CDK inhibitors (CKIs), which negatively modulate the cell cycle progression, also participate in regulating the activity of CDKs (31). For example, p21, p27, and p57, three important CKIs of the Cip/Kip family, function to inhibit the cell cycle progression by binding and inhibiting the cyclin E-CDK2 complex (25). Moreover, p21 can directly bind to the proliferating cell nuclear antigen (PCNA), a DNA polymerase activity factor, to inhibit DNA synthesis, thereby impeding cell progression (32). Although the cell cycle is strictly regulated, a growing number of viruses have been demonstrated to evolve diverse strategies of utilizing the components of cell cycle to facilitate their own replication (33–37).

In the present study, we first demonstrated that PRRSV infection can cause p21 degradation and promote MARC-145 cells entry into the cell cycle's S phase, both of which are beneficial to PRRSV replication. By means of constructing a p21-knockout MARC-145 cell line using CRISPR/Cas9 technology, the inhibitory effect of p21 on PRRSV replication was confirmed. In addition, we further demonstrated the PRRSV nsp11 protein mediate the degradation of p21 in a ubiquitin-independent and proteasome-dependent degradation manner, which required the endoribonuclease activity of nsp11.

## Materials and Methods

### Cells, Virus, Antibodies, and Plasmid

MARC-145 and 293FT cells were cultured in Dulbecco's modified Eagle's medium (DMEM, Gibco) supplemented with 10% fetal bovine serum (FBS, Gibco). Porcine pulmonary alveolar macrophages (PAMs) were prepared as previously described (38), and cultured in RPMI 1640 medium containing 10% FBS. All cells were cultured at 37°C with 5% CO_2_ in a humidified incubator (Thermo Fisher Scientific). The Chinese highly pathogenic PRRSV (HP-PRRSV) strain JXwn06 (GenBank accession no. EF641008) and low pathogenic PRRSV (LP-PRRSV) strain HB-1/3.9 (EU360130) were used in this study (39). Rabbit anti-p21 and anti-p27 polyclonal antibodies (pAb) were purchased from Proteintech Group (Chicago, IL, USA). Mouse anti-β-actin monoclonal antibody (mAb), mouse anti-HA mAb, rabbit anti-Myc pAb, mouse anti-Flag mAb were all purchased from Sigma-Aldrich. The specific mAbs raised against the N and nsp11 proteins of PRRSV were prepared in our laboratory. The plasmid pcDNA3.1-HA-Ubiquitin was purchased from Bioworld Technology, Inc. (Bloomington, MN, USA).

### Plasmid Construction and Transfection

The p21 protein-coding gene was amplified from PAMs by reverse transcription-polymerase chain reaction (RT-PCR) using two sets of primer pairs listed in Table 1. The resulting amplicons obtained with primer pairs HA/Myc-p21-F and HA/Myc-p21-R were cloned into vectors pCMV-HA (Clontech) and pCMV-Myc (Clontech) between the EcoRI and KpnI sites to generate recombinant plasmids pCMV-HA-p21 and pCMV-Myc-p21, which will produce an HA-tagged and an Myc-tagged p21 protein, respectively. The resulting amplicon obtained with primer pair Flag-p21-F and Flag-p21-R was cloned into vector p3xFLAG-CMV™-10 (Sigma-Aldrich) between the EcoRI and KpnI sites to generate a recombinant plasmid pCMV-Flag-p21, which will produce a Flag-tagged p21 protein. The eukaryotic plasmids expressing each individual protein of PRRSV JXwn06, including pCMV-HA-nsp1α, pCMV-HA-nsp1β, pCMV-HA-nsp2, pCMV-HA-nsp3, pCMV-HA-nsp4, pCMV-HA-nsp5, pCMV-HA-nsp8, pCMV-HA-nsp9, pCMV-HA-nsp10, pCMV-HA-nsp11, pCMV-Myc-nsp11, pCMV-HA-nsp12, pCMV-HA-OFR5, pCMV-HA-ORF6, pCMV-HA-ORF7, were prepared in our laboratory as described previously (40–43). Moreover, four mutants of plasmid pCMV-HA-nsp11 were constructed using a fast site-directed mutagenesis kit (TransGen Biotech Co., Ltd, Beijing, China) according to the manufacturer's instructions and with the respective mutagenic primer pairs listed in Table 1. The resulting four mutant plasmids, designated M1, M2, M3, and M4, contained a mutation at the position of 112 (C → A), 129 (H → A), 144 (H → A), and 173 (K → A) amino acids of nsp11 protein, respectively. All the constructed plasmids were verified by DNA sequencing to ensure their accuracy. Transfection of cells with the constructed plasmids was performed using Lipofectamine™ LTX Reagent (Invitrogen) according to the manufacturer's instructions.

**Table 1:** Primers used in this study.

Primers*	**Sequence (5^′^→3^′^)*^a^***	**Application**	**Restriction enzymes**
HA/Myc-p21-F	CCG*GAATTC*GGATGTCAGAGTCGACCAG	p21 gene amplification and pCMV-HA/Myc-p21 construction	*EcoR I*
HA/Myc-p21-R	CGG*GGTACC*TTAGGGCTTCCTCTTGGAG		*Kpn I*
Flag-p21-F	CCG*GAATTC*AATGTCAGAGTCGACCAG	pCMV-Flag-p21 construction	*EcoR I*
Flag-p21-R	CGG*GGTACC*GATTAGGGCTTCCTCTTGGAG		*Kpn I*
HA-Nsp11-C112A-F	GAATTGAGGTAGAT**GC**TCGAGAGTATC	M1 construction	
HA-Nsp11-C112A-R	**GC**ATCTACCTCAATTCGGCCGGTGCT		
HA-Nsp11-H129A-F	CTGAGTCCCTCCCA**GC**TGCCTTCATC	M2 construction	
HA-Nsp11-H129A-R	**GC**TGGGAGGGACTCAGCAACTTCTCG		
HA-Nsp11-H144A-F	CCGTTGGGGGATGT**GC**TCACGTTACC	M3 construction	
HA-Nsp11-H144A-R	**GC**ACATCCCCCAACGGTGGTACCTTT		
HA-Nsp11-K173A-F	CCGGGAAAGCCGCG**GC**AGCAGTTTGC	M4 construction	
HA-Nsp11-K173A-R	**GC**CGCGGCTTTCCCGGGGCTCGAAAC		
p21sgRNA#1-F	CACCGCCGCGACTGTGATGCGCTAA	pX335- p21sgRNA#1 construction	
p21sgRNA#1-R	AAACTTAGCGCATCACAGTCGCGGC		
p21sgRNA#2-F	CACCGCGCTGTCCACTGGGCCGAAG	pX335- p21sgRNA#2 construction	
p21sgRNA#2-R	AAACCTTCGGCCCAGTGGACAGCGC		
pWPXL-nsp11-F	GG*GTTTAAAC*TAATGGGGTCGAGCTC	pWPXL-nsp11 construction	*PmeI*
pWPXL-nsp11-R	CG*ACGCGT*AACTATTCAAGTTGAAAATAG		*MluI*
p21-F	CCATGTGGACCTGTCGCTAT	p21 real-time PCR	
p21-R	CGGCGTTTGGAGTGGTAGAA		
β-actin-F	TCCCTGGAGAAGAGCTACGA	β-actin real-time PCR	
β-actin-R	AGCACTGTGTTGGCGTACAG		
p21 knockout-F	ATTCACACCCATGAGGGAC	DNA sequencing of p21-knockout MARC-145 cells	
p21 knockout-R	CTGTCATGCTGGTCTG		

a *The nucleotides in the primer sequences used for introducing a single point mutation into the PRRSV nsp11 are marked in bold*.

### Virus Infection and Titration

MARC-145 cells grown to ~90% confluence were infected with the HP-PRRSV strain JXwn06 at a multiplicity of infection (MOI) of 0.01, 0.1, or 1, or with the LP-PRRSV strain HB-1/3.9 at an MOI of 1, or mock infected with DMEM. After adsorption for 1 h at 37°C with 5% CO_2_ atmosphere, the viral inoculum was removed and the cells were washed twice with sterile phosphate-buffered saline (PBS). The cells were then cultured in fresh DMEM containing 5% FBS until they reached the specified time points required for the subsequent assays. Viral titers were determined by measuring the 50% tissue culture infectious dose (TCID_50_) using a microtitration infectivity assay as previously described (39).

### Western Blot

Cell samples were rinsed thrice with PBS and then lysed with Pierce™ IP lysis buffer (Thermo Scientific) supplemented with protease inhibitor cocktail (Sigma-Aldrich). After centrifugation for 20 min at 12,000 g and 4°C, the supernatant was used as the total cellular proteins, whose concentration was determined using a Pierce BCA protein assay kit (Thermo Scientific). Approximately 20 μg of each sample was subjected to western blot analyses. Briefly, protein samples were separated on SDS-PAGE gels and then electrically transferred onto 0.22 μm polyvinylidene difluoride (PVDF) membranes (Millipore). The membranes were blocked with 5% skim milk diluted in PBST (PBS with 0.05% Tween-20) for 1.5 h at room temperature, and then probed with appropriate primary antibodies for 1 h at 37°C. After three washes with PBST, the membranes were incubated with the corresponding horseradish peroxidase (HRP)-conjugated secondary antibodies for 1 h at 37°C. The target protein signals were developed using the Pierce™ ECL western blotting substrate (Thermo Scientific), and images were taken with a ProteinSimple FluorChem E image system (Santa Clara, CA, USA).

### RNA Interference

Three sets of small interfering RNAs (siRNAs; Si-215, Si-462, and Si-432) targeting different regions of the p21 gene were designed to specifically knock down p21 gene expression in MARC-145 cells (Table 2). The p21-specific siRNAs along with the scrambled siRNAs (Si-NC) were synthesized by the GenePharma (Suzhou, China). MARC-145 cells grown to ~40% confluence in 6-well cell culture plates (Corning) were transfected with the synthesized siRNAs using Lipofectamine™ RNAiMAX reagent (Invitrogen) as per the manufacturer's protocol. At the indicated time points post-transfection, the cells were harvested for analysis of silencing efficiency and its influence on the cell cycle.

**Table 2:** The designed p21-specific and scrambled siRNAs for knockdown of p21 protein in MARC-145 cells.

**siRNAs**	**Sequence (5^′^→3^′^)**
Si-215	GCGACUGUGAUGCGCUAAUTT
	AUUAGCGCAUCACAGUCGCTT
Si-432	GACAGCAGAGGAAGACCAUTT
	AUGGUCUUCCUCYGCUCUCTT
Si-462	GCUAUCUUGUACCCUUGUGTT
	CACAAGGGUACAAGAUAGCTT
Si-NC	UUCUCCGAACGUGUCACGUTT
	ACGUGACACGUUCGGAGAATT

### Flow Cytometry Analysis of Cell Cycle

Cell cycle distribution was determined by DNA flow cytometric analysis. Briefly, mock- or PRRSV-infected MARC-145 cells grown in 12-well plates at ~5 × 10^5^ cells/well were harvested by trypsinization, collected by centrifugation (200 g, 5 min), and washed twice with cold PBS. The cells were then fixed with cold 70% ethanol overnight at 4°C. After another washing step with cold PBS, the cell pellets were resuspended and stained with 500 μl of propidium iodide (PI) staining buffer (BD Biosciences, San Jose, CA, USA) containing 0.5 mg/ml RNase A for 15 min at room temperature in the dark. The cell cycle phases were determined by measuring DNA contents using a FACSCalibur flow cytometer (BD Biosciences), and the data were analyzed using the ModFit LT™ software Version 3.1 (Verity Software House, Topsham, ME, USA).

### Cell Cycle Synchronization

For synchronization of the G0/G1 phase, a serum-deprivation method was used to treat MARC-145 cells (44). Briefly, when MARC-145 cells grown in 6-well cell culture plates with DMEM containing 10% FBS reached ~30% confluence, the medium was discarded and the cells were washed twice with sterile PBS. The cells were starved for 36 h in FBS-free DMEM and then harvested for G0/G1 phase analysis. For synchronization of the S phase, a double-thymidine block method was employed to treat MARC-145 cells (45). In brief, when MARC-145 cells grown in 6-well plates with DMEM containing 10% FBS approached ~30% confluence, the medium was discarded and the cells were washed twice with sterile PBS. Fresh DMEM containing 10% FBS and 2 mM thymidine (Sigma-Aldrich) was added to the cells and then cultured for 16 h. After another washing step, refresh DMEM containing 10% FBS without thymidine was added to cells and then cultured for 8 h. After a final washing step, the cells were cultured in fresh DMEM containing 10% FBS and 2 mM thymidine for an additional 16 h and then harvested for S phase analysis. For synchronization of the G2/M phase, nocodazole block was used to treat MARC-145 cells (25). Briefly, MARC-145 cells reaching 80–90% confluence were washed as described above and then cultured for 24 h in refresh DMEM containing 10% FBS and 60 ng/ml nocodazole (Sigma-Aldrich). The treated cells were collected for G2/M phase analysis.

### Construction of p21 Gene-Knockout MARC-145 Cells

The system of a Cas9 mutant D10A nickase together with a pair of sgRNAs were used to establish p21 gene-knockout MARC-145 cells using a previously described protocol (46). Briefly, the p21 gene-specific sgRNAs were designed using the online CRISPR design tool (http://crisp.mit.edu) and were shown as follows: p21sgRNA#1: 5′-CCGCGACTGTGATGCGCTAA-3′, p21sgRNA#2: 5′-CGCTGTCCACTGGGCCGAAG-3′. After annealing with two pairs of corresponding primers (p21sgRNA#1-F/R and p21sgRNA#2-F/R) shown in Table 1, two double-stranded oligonucleotides of sgRNAs were obtained, and then they were separately inserted into the BbsI sites of pX335 vector, which contains the expression cassettes for hCas9 (D10A), to generate recombinant plasmids pX335-p21sgRNA#1 and pX335-p21sgRNA#2 using the method described by Cong et al. (47). After verification by DNA sequencing, MARC-145 cells grown in a 10-cm dish at 80–90% confluence were transfected with 10 μg of pX335-p21sgRNA#1, 10 μg of pX335-p21sgRNA#2 and 1 μg of pEGFP-N1 vector (Clontech) using Lipofectamine™ LTX Reagent (Invitrogen) according to the manufacturer's protocol. At 24 h post-transfection (hpt), the cells were dissociated into single cells by trypsin digestion and resuspended in fresh DMEM. The dispersed cells were sorted into 96-well plates by fluorescence-activated cell sorting (FACS) using a MoFlo™ XDP cell sorter (Beckman Coulter, CA, USA) gated on EGFP expression under sterile conditions. Following a period of cultivation and expansion, cellular genomic DNA was extracted from the obtained MARC-145 cells using the TIANamp Genomic DNA Kit (TIANGEN Biotech Co., Ltd, Beijing, China). To confirm whether the p21 gene had been successfully knocked out from the cells, PCR was performed on the extracted genomic DNA to amplify the genomic fragment encompassing the CRISPR/Cas9 target sequence using primer pair p21 knockout-F/R (Table 1). The amplicon was subjected to DNA sequencing. Moreover, western blot analysis was also carried out to further confirm the successful knockout of p21 gene from the obtained MARC-145 cells.

### Construction of MARC-145 Cells Constitutively Expressing the PRRSV nsp11 Protein

The lentiviral expression system, consisting of three vectors pWPXL, pMD2.G and psPAX2 whose information are available on the Addgene website (http://www.addgene.org/), was used to construct nsp11-overexpressing MARC-145 cells. The pWPXL vector we used was modified by replacing the GFP tag with a Flag tag in our laboratory as previously described, and was designated pWPXL-Flag (48). The PCR-amplified fragment containing the complete nucleotide sequence of nsp11 was cloned into the modified vector pWPXL-Flag between PmeI and MluI restriction sites using the primer pair pWPXL-nsp11-F/R (Table 1). The resulting recombinant plasmid pWPXL-nsp11 along with pMD2.G and psPAX2 were co-transfected into 293FT cells to rescue recombinant lentiviruses using the FuGENE® HD Transfection Reagent (Roche Applied Science, Indianapolis, IN, USA) as we previously described (48). Infectious lentiviruses were harvested by collecting culture supernatants when the transfected cells exhibited obvious cytopathic effects, and filtered through 0.45-μm filters. The filtered lentiviruses were then used to transduce MARC-145 cells in the presence of 8 μg/ml polybrene (Sigma-Aldrich) as previously described (49).

### RNA Extraction and Real-Time PCR Assay

Total cellular RNAs of mock- or PRRSV-infected MARC-145 cells or cells co-transfected with plasmids pCMV-Myc-p21 and pCMV-HA-nsp11 or its derivative mutants were extracted using the RNAprep pure Cell Kit (Tiangen Biotech Co., Ltd, Beijing, China) according to the manufacturer's protocol. The first strand cDNA was synthesized using 1 μg of cellular RNAs with a FastQuant RT Kit (Tiangen Biotech Co., Ltd) following the manufacturer's instructions. To determine the mRNA expression levels of p21, real-time PCR assays were performed using a TaKaRa SYBR Premix Ex Taq kit on an ABI 7500 real-time PCR system along with β-actin as a housekeeping gene as previously described (50). The corresponding primers (p21-F/R and β-actin-F/R) were listed in Table 1. Fold-change values were calculated according to the 2^−ΔΔCt^ method and normalized to the β-actin reference gene (51).

### Immunoprecipitation and Ubiquitination Assays

To determine the ubiquitination status of p21 protein in the presence of wild-type nsp11 protein, 293FT cells grown to 70–80% confluence in 6-well cell plates were co-transfected with 1 μg of pcDNA3.1-HA-Ubiquitin, 1 μg of pCMV-Flag-p21 and 1 μg of pCMV-Myc-nsp11 by using Lipofectamine LTX reagent according to the manufacturer's instruction. At 20 hpt, the cells were treated with 10 μM of MG132 (Selleck Chemicals LLC, Houston, TX, USA) for 4 h and then lysed with IP buffer (Beyotime Biotechnology, Shanghai, China) containing protease inhibitor cocktail (Sigma-Aldrich). After centrifugation for 20 min (4°C, 12000 rpm), the cell lysates were precleared with 20 μl of protein A/G Sepharose beads (GE Healthcare, Boston, MA, USA) by shaking at 4°C for 30 min. After centrifugation for 1min (4°C, 12000 rpm), the supernatants were precipitated with 1 μg of anti-Flag mAb and 20 μl of protein A/G Sepharose beads by shaking overnight at 4°C. The beads were washed five times with IP buffer and one time with ultrapure water, the immune complexes were separated on 12% SDS-PAGE gels and then analyzed by western blot analysis.

### Statistical Analysis

Statistical significance between different groups was analyzed by two-way analysis of variance (ANOVA) test using GraphPad Prism software (Version 5.0; La Jolla, CA, USA). Data were presented as means ± standard deviations (SD), and differences with a value of *p* < 0.05 were considered statistically significant. The asterisks indicate the statistical significance: ^*^p < 0.05; ^**^p < 0.01; ^***^p < 0.001.

## Results

### PRRSV Infection Downregulates p21 Protein Expression in MARC-145 Cells

**Figure 1:**
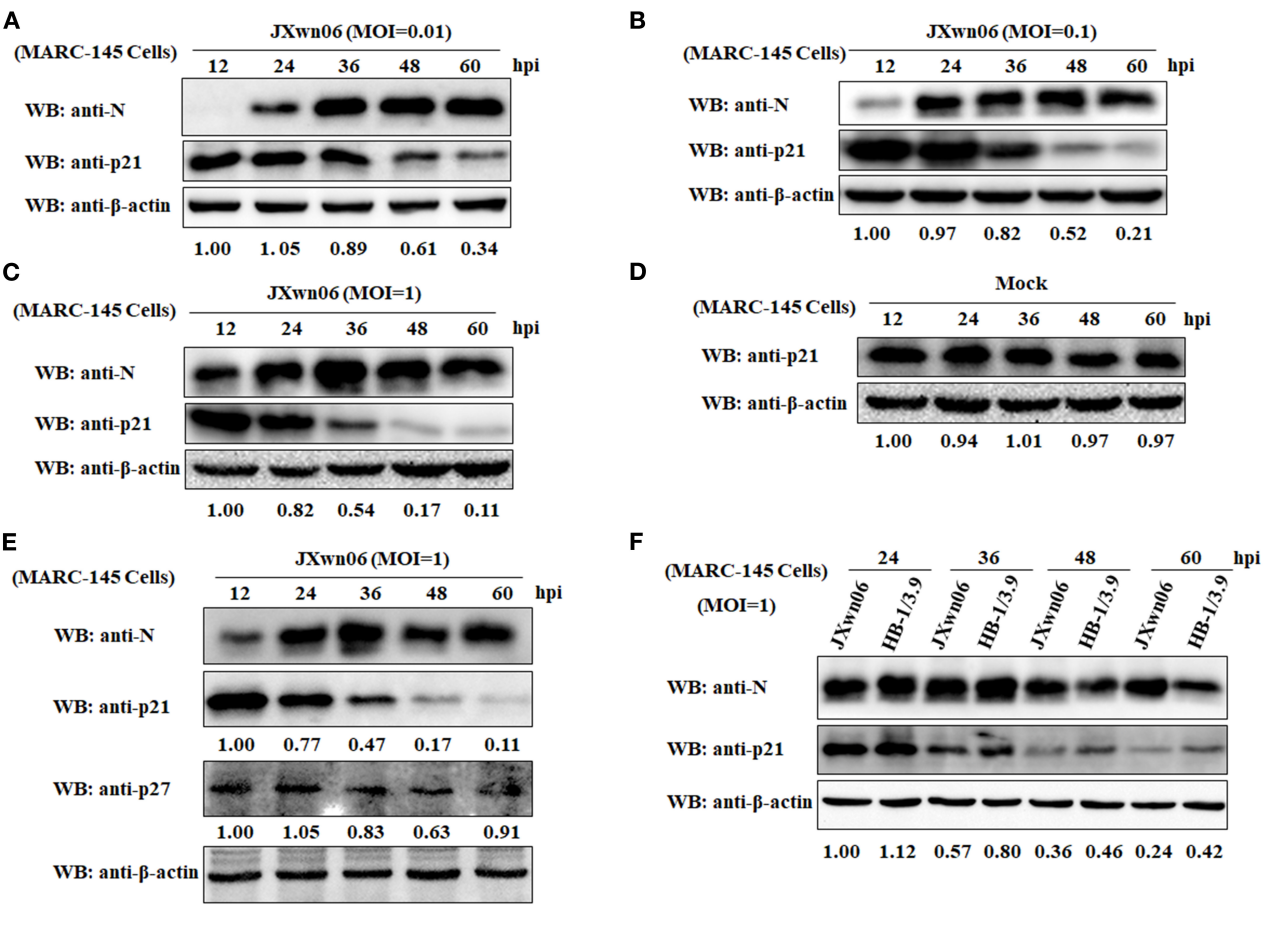
PRRSV infection downregulates p21 protein expression in MARC-145 cells. **(A)** MARC-145 cells were infected with the HP-PRRSV strain JXwn06 at a low MOI of 0.01. At 12, 24, 36, 48, and 60 hpi, the cell samples were harvested and processed for western blot analysis using rabbit anti-p21 pAb and mouse anti-PRRSV N mAb. β-Actin was used as a protein loading control. The densitometry ratios of p21/β-actin are indicated below the corresponding protein bands. **(B)** MARC-145 cells were infected with the HP-PRRSV strain JXwn06 at a medium MOI of 0.1, and then harvested, processed and analyzed as in **(A)**. **(C)** MARC-145 cells were infected with the HP-PRRSV strain JXwn06 at a high MOI of 1, and then harvested, processed and analyzed as in **(A)**. **(D)** MARC-145 cells were mock infected with DMEM, and then harvested, processed and analyzed as in **(A)**. **(E)** MARC-145 cells were infected, harvested, processed and analyzed as in **(C)** but with an additional pAb against p27 protein. **(F)** MARC-145 cells were infected with the HP-PRRSV strain JXwn06 or the LP-PRRSV strain HB-1/3.9 at an MOI of 1. At 24, 36, 48, and 60 hpi, the cell samples were harvested, processed and analyzed as in **(A)**.

To elucidate whether PRRSV infection causes changes of cell cycle progression, MARC-145 cells were mock infected or infected with the HP-PRRSV strain JXwn06 at three MOIs (0.01, 0.1, and 1) or the LP-PRRSV strain HB-1/3.9 at an MOI of 1. Protein samples were prepared from harvested cells at increasing intervals of time following infection, and were subjected to western blot analyses for a well-known CDK inhibitor of the cell cycle—p21 protein (25, 52). A mAb that specifically recognizes the N protein of PRRSV was used to track the progression of viral infection. As shown in Figures 1A–C, the expression levels of cellular p21 protein in JXwn06-infected MARC-145 cells gradually decreased as the infection progressed, and the change trend was more obvious from 36 h post-infection (hpi) onward. Additionally, of the three MOIs tested, MOI of 1 exhibited the highest inhibition effect on p21 expression (Figures 1A–C). This indicates that the effect of PRRSV infection on p21 protein expression was in a dose-dependent manner. In contrast, the expression of p21 protein in the mock-infected MARC-145 cells underwent no obvious changes within the same time points (Figure 1D). In addition to p21, another important member of the CDK inhibitors—p27 protein—was also determined, but no obvious change in protein level was observed at the selected time points post PRRSV infection (Figure 1E). Furthermore, we also tested the effect of the LP-PRRSV strain HB-1/3.9 on the expression of p21 protein in MARC-145 cells using the high MOI of 1. As shown in Figure 1F, HB-1/3.9 was also able to cause downregulation of p21 in MARC-145 cells, but to a lesser extent than did the HP-PRRSV strain JXwn06. Taken together, these results indicate that PRRSV infection caused downregulation of p21 protein in MARC-145 cells in a dose-dependent manner.

### Knockdown of p21 Promotes MARC-145 Cells Entry Into the S Phase of Cell Cycle

Because p21 can induce cell cycle arrest at the G1 phase and thus block cells entry into the S phase by inactivating CDKs or by inhibiting activity of PCNA (52), the downregulation of p21 protein might be indicative of promotion of S-phase entry. To address this, we designed three p21-specific siRNAs (Si-215, Si-432, and Si-462) aiming to specifically silence p21 expression in MARC-145 cells. To obtain the optimal silence efficiency, three doses (20, 30, and 40 pmol/well) of each siRNAs were transfected into MARC-145 cells grown in 6-well cell plates. At 36 hpt, the transfected cells were harvested and processed for western blot analyses. As shown in Supplementary Figure 1, MARC-145 cells transfected with Si-215 or Si-462 siRNAs exhibited a significantly reduced level of endogenous p21 protein, as compared to the cells transfected with Si-432 or the scrambled siRNAs (Si-NC). To be specific, Si-215 displayed the best silencing effect among the two effective siRNAs, which resulted in a nearly 90% reduction of p21 protein expression. Furthermore, no significant difference was observed between the three transfection doses. Therefore, Si-215 siRNAs at a working dose of 20 pmol/well were chosen for the subsequent experiments.

**Figure 2:**
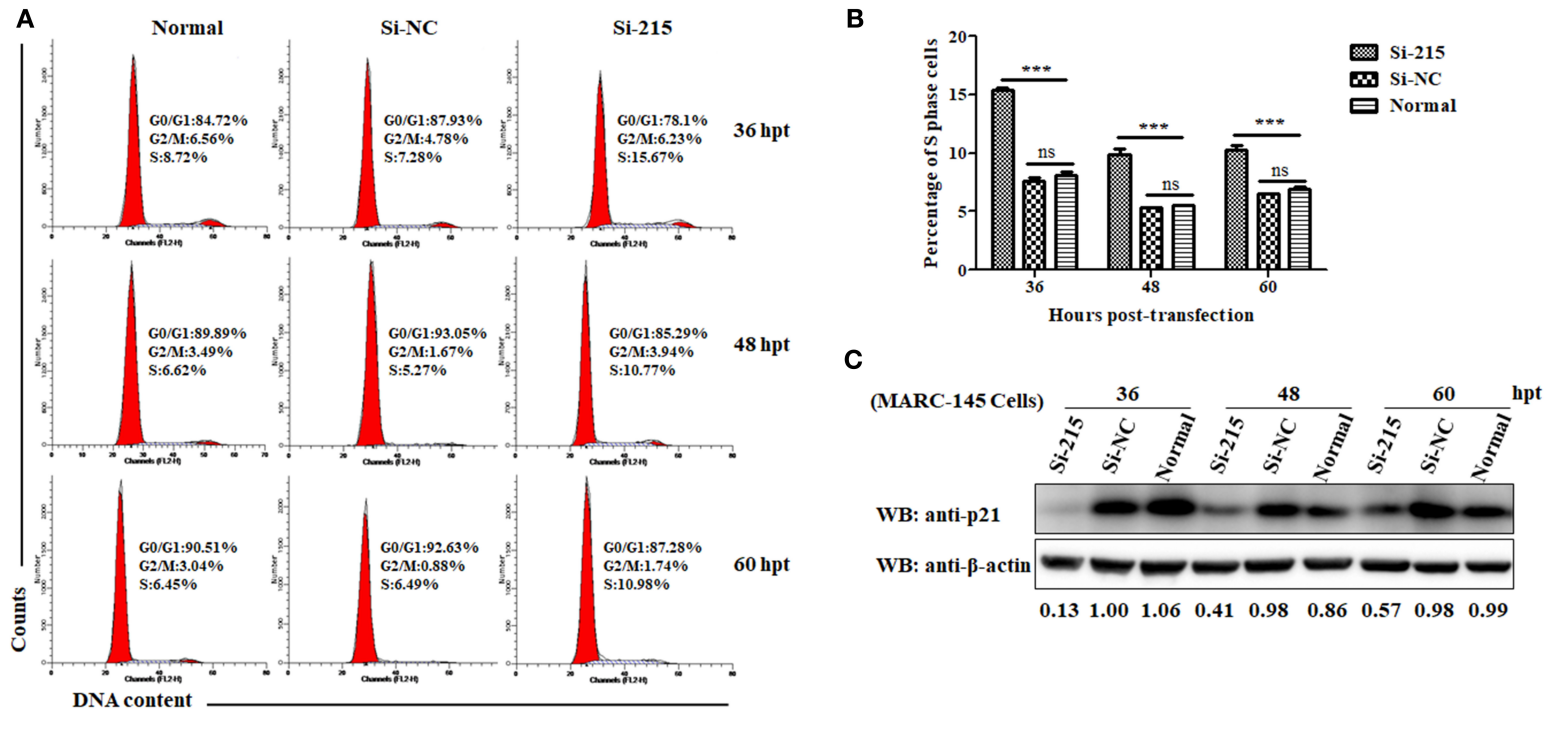
Knockdown of p21 protein promotes MARC-145 cells entry into the S phase of cell cycle. **(A)** Flow cytometry analysis of cell cycle using PI staining of nuclear DNA. Normal MARC-145 cells and cells transfected with p21-specific siRNAs (Si-125) or scrambled siRNAs (Si-NC) were harvested for cell cycle phase analysis at 36, 48, and 60 hpt. G0/G1: Gap 0 and Gap 1 phases; S: synthesis phase; G2/M: Gap 2 and mitotic phases. Representative results are shown and similar results were obtained in three independent experiments. **(B)** Statistical analysis of S-phase cell numbers of normal MARC-145 cells or cells transfected with Si-125 or Si-NC siRNAs at 36, 48, and 60 hpt. Data are expressed as means ± SD of three independent experiments (two-way ANOVA test; ***p < 0.001; ns, no significant difference). **(C)** Normal MARC-145 cells and cells transfected with Si-125 or Si-NC siRNAs at 36, 48, and 60 hpt were harvested for western blot analyses using a pAb against p21 protein. β-Actin was used as a protein loading control. The densitometry ratios of p21/β-actin are indicated below the corresponding protein bands.

To analyze the effect of p21 knockdown on cell cycle progression, flow cytometry was performed on normal MARC-145 cells or cells transfected with Si-215 or Si-NC siRNAs by determining the changes of nuclear DNA content using PI staining. As shown in Figures 2A,B, the S-phase cell numbers of Si-215-transfected MARC-145 cells were significantly higher than those of the normal or Si-NC-transfected MARC-145 cells at 36, 48, and 60 hpt. Interestingly, the S-phase cell numbers of Si-215-transfected MARC-145 cells at 36 hpt were higher than those at 48 and 60 hpt. To find a reasonable explanation for this phenomenon, we detected the expression levels of p21 protein in normal MARC-145 cells or cells transfected with Si-215 or Si-NC siRNAs by western blot analyses. As shown in Figure 2C, the expression level of p21 protein in Si-215-transfected MARC-145 cells at 36 hpt was the lowest among the three time points post-transfection. Accordingly, we speculate that the S-phase cell numbers of Si-215-transfected MARC-145 cells were negatively correlated with the expression level of p21 protein. Altogether, these results suggest that RNA interference-mediated knockdown of p21 promotes MARC-145 cells entry into the S phase of cell cycle.

### PRRSV Infection Promotes the Entry of MARC-145 Cells Into the S Phase of Cell Cycle

**Figure 3:**
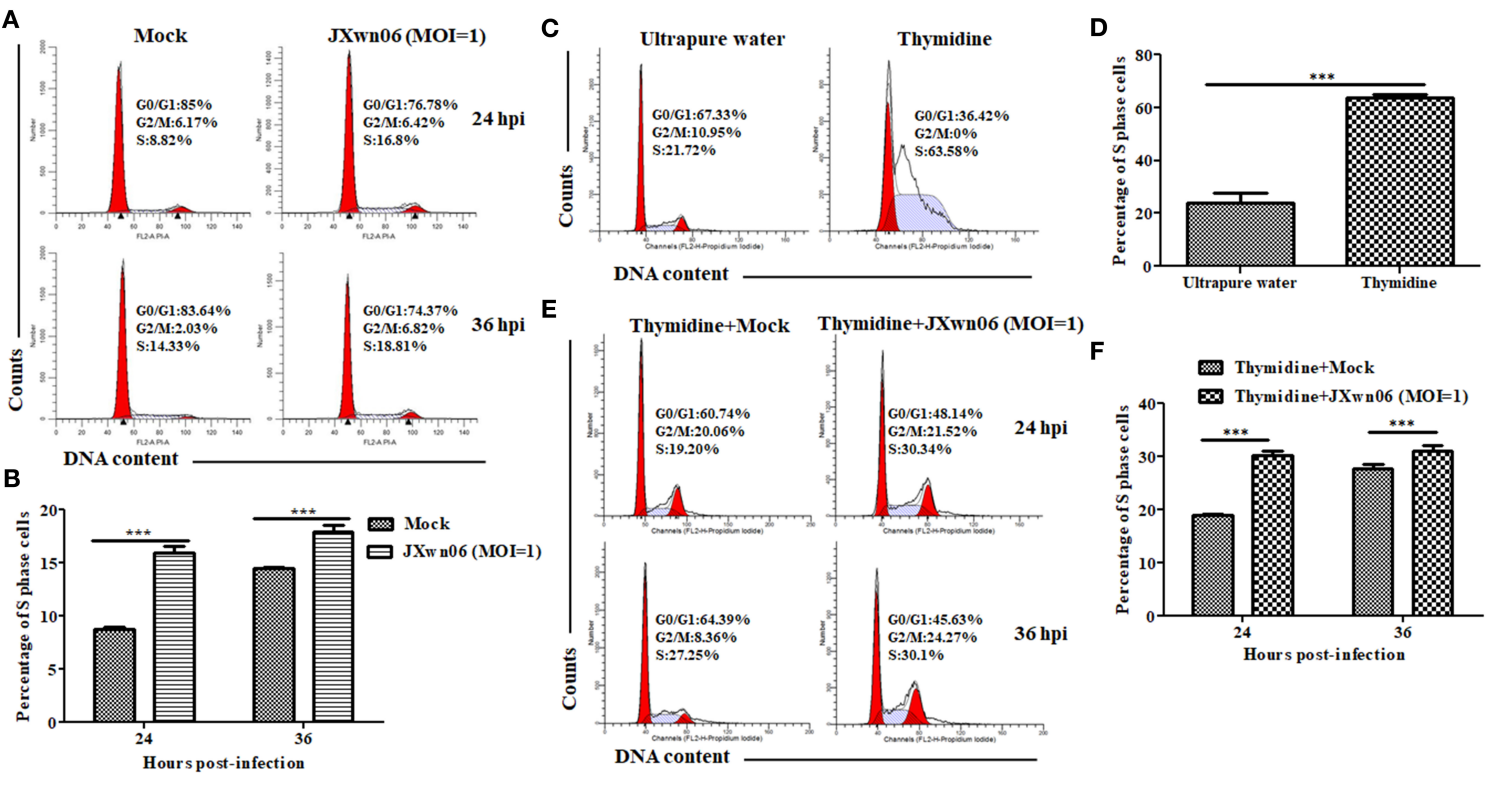
PRRSV infection promotes the entry of MARC-145 cells into the S phase of cell cycle. **(A)** Flow cytometry analysis of cell cycle using PI staining of nuclear DNA. MARC-145 cells grown to ~90% confluence were mock infected or infected with the HP-PRRSV strain JXwn06 at an MOI of 1, and then harvested for cell cycle phase analysis at 24 and 36 hpi. G0/G1: Gap 0 and Gap 1 phases; S: synthesis phase; G2/M: Gap 2 and mitotic phases. Representative results are shown and similar results were obtained in three independent experiments. **(B)** Statistical analysis of S-phase cell numbers of mock- and JXwn06-infected MARC-145 cells at 24 and 36 hpi. Data are expressed as means ± SD of three independent experiments (two-way ANOVA test; ***p < 0.001). **(C)** Cell cycle phase analyses of MARC-145 cells treated by double-thymidine block or by ultrapure water. **(D)** Statistical analysis of S-phase cell numbers of MARC-145 cells treated by double-thymidine block or by ultrapure water. Data are expressed as means ± SD of three independent experiments (two-way ANOVA test; ***p < 0.001). **(E)** Cell cycle phase analyses of mock- and JXwn06-infected MARC-145 cells that were pretreated with double thymidine. **(F)** Statistical analysis of S-phase cell numbers of mock- and JXwn06-infected MARC-145 cells that were pretreated with double thymidine. Data are expressed as means ± SD of three independent experiments (two-way ANOVA test; ***p < 0.001).

Having demonstrated that PRRSV infection downregulates p21 expression and knockdown of p21 promotes cells entry into S phase in MARC-145 cells, we proceeded to analyze the effect of PRRSV infection on cell cycle progression using double thymidine-treated MARC-145 cells as the S-phase synchronization control (45). As shown in Figures 3A,B, the S-phase cell numbers of JXwn06-infected MARC-145 cells were significantly higher than those of the mock-infected cells at 24 and 36 hpi, which is consistent with the double thymidine-treated cells that were synchronized in S phase (Figures 3C,D). These data indicate that PRRSV infection was able to promote cells entry into the S phase. For further confirmation, MARC-145 cells were first synchronized into S phase by double-thymidine treatment and then infected with JXwn06 at an MOI of 1 for 24 and 36 h. Flow cytometric analysis of nuclear DNA contents demonstrated that double-thymidine block synchronization further increased the S-phase cell numbers of JXwn06-infected MARC-145 cells (Figures 3E,F), as compared to the JXwn06-infected MARC-145 cells without thymidine treatment (Figures 3A,B). Collectively, these results suggest that PRRSV infection promotes the entry of MARC-145 cells into the S phase of cell cycle.

### MARC-145 Cells in S Phase or p21 Knockout Cells Are Conducive to PRRSV Replication

**Figure 4:**
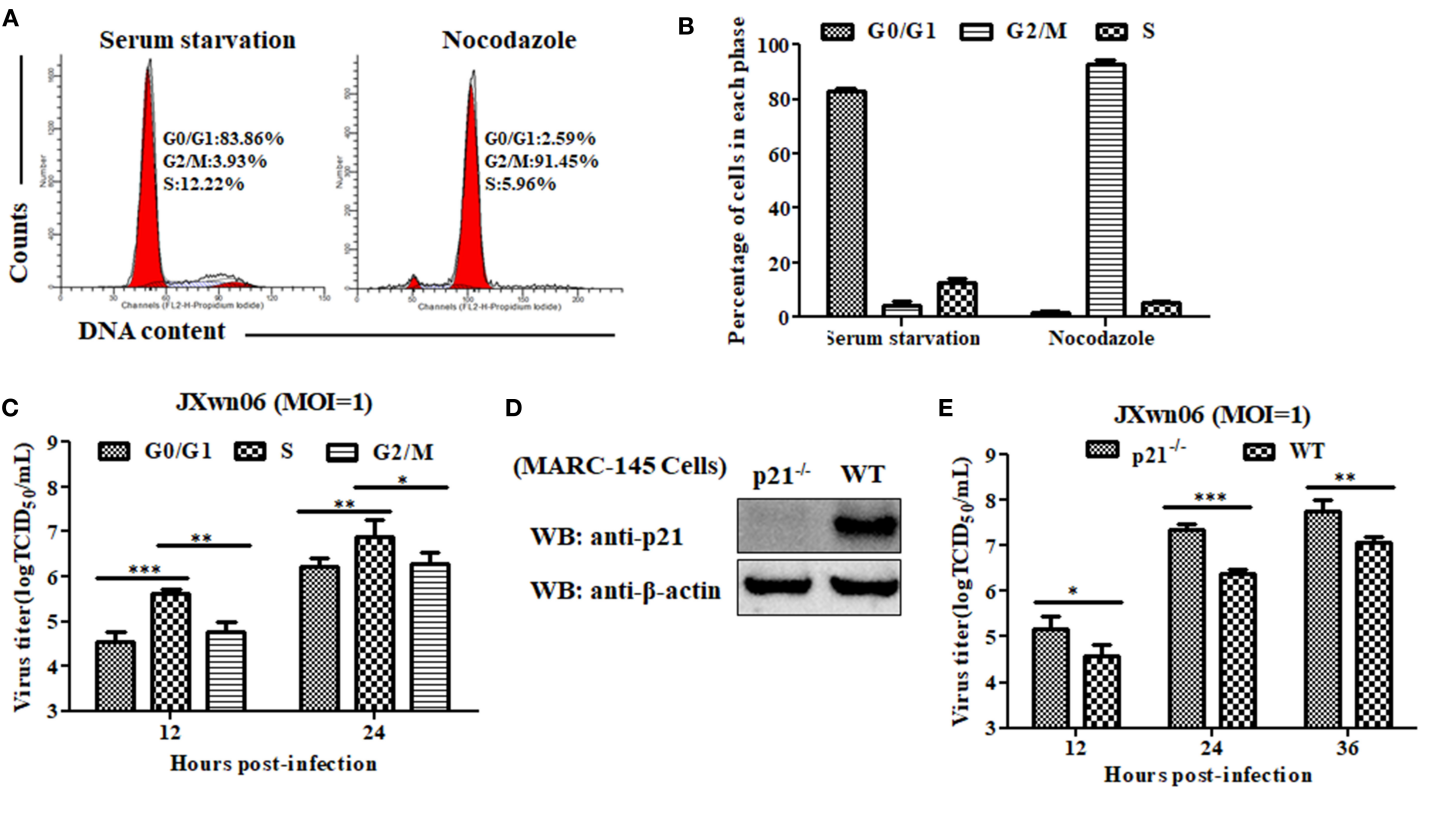
MARC-145 cells in S phase or without p21 protein are conducive to PRRSV replication. **(A)** Flow cytometry analysis of cell cycle using PI staining of nuclear DNA. MARC-145 cells were treated by serum deprivation or nocodazole, and then harvested for cell cycle phase analysis. G0/G1: Gap 0 and Gap 1 phases; S: synthesis phase; G2/M: Gap 2 and mitotic phases. Representative results are shown and similar results were obtained in three independent experiments. **(B)** Statistical analysis of cell numbers in each phase of MARC-145 cells treated with serum deprivation or nocodazole. Data are expressed as means ± SD of three independent experiments (two-way ANOVA test; ***p < 0.001). **(C)** MARC-145 cells synchronized in G0/G1, S and G2/M phases were infected with PRRSV JXwn06 at a MOI of 1 for 12 and 24 h. The viral titers were determined by TCID_50_ assay. Data are expressed as means ± SD of three independent experiments (two-way ANOVA test; *p < 0.05; **p < 0.01; ***p < 0.001). **(D)** Western blot analysis of endogenous p21 protein in p21-knockout MARC-145 cells and wild-type cells. **(E)** Comparison of the capability of p21-knockout MARC-145 cells and wild-type cells to support PRRSV replication. Cells were infected with PRRSV JXwn06 at a MOI of 1 for 12, 24 and 36 h. The viral titers were determined by TCID_50_ assay. Data are expressed as means ± SD of three independent experiments (two-way ANOVA test; *p < 0.05; **p < 0.01; ***p < 0.001).

To explore what role of each cell-cycle phase plays in the replication of PRRSV, we first synchronized MARC-145 cells into G0/G1, S and G2/M phases using serum-deprivation (44), double-thymidine block (45), and nocodazole treatment methods (25), respectively, and then evaluated the capability of these cells in different phases to support PRRSV replication. As shown in Figures 3C,D, 4A,B, MARC-145 cells were successfully synchronized into G0/G1, S and G2/M phases. Subsequently, these cells in different phases were infected with JXwn06 at an MOI of 1. At 12 and 24 hpi, the yield of progeny viruses was determined by TCID_50_ assay. As shown in Figure 4C, the viral titers of MARC-145 cells synchronized into S phase were statistically higher than those of the other two synchronized cells in G0/G1 and G2/M phases. These data reveal that MARC-145 cells in S phase are beneficial to PRRSV replication.

Although we have preliminarily proven that RNA interference-mediated knockdown of p21 can promote MARC-145 cells entry into the S phase of cell cycle, low levels of p21 protein still exist in the cells upon transfection with Si-215 siRNAs (Figure 2 and Supplementary Figure 1), which might affect the reliability of the results. For further confirmation, we took a step further by constructing p21 gene-knockout MARC-145 cell lines using the CRISPR/Cas9 system along with a pair of sgRNAs (46). Their positions within the p21 gene are indicated in Supplementary Figure 2A. Via flow cytometry screening, one of the generated cell lines was picked out and designated p21^−/−^. DNA sequencing of the amplified genomic region encompassing the CRISPR/Cas9 target sequence showed that there was a 13-bp insertion in the targeted locus of p21^−/−^ cells (Supplementary Figure 2B), indicating that the open reading frame coding for p21 protein was broken. Furthermore, western blot analysis was performed on total cell lysates of p21^−/−^ cells using rabbit anti-p21 pAb, but no p21 protein was detectable in the cells (Figure 4D). These results suggest that p21 was successfully knocked out in p21^−/−^ cells. On this basis, the capability of p21^−/−^ cells to support PRRSV replication was evaluated by virus infection and subsequent determination of progeny virus yields. As shown in Figure 4E, the virus titers in p21^−/−^ cells were significantly higher than those of the wild-type MARC-145 cells at 12, 24, and 36 hpi. This indicates that knockout of p21 protein is conducive to PRRSV replication in MARC-145 cells.

### The PRRSV nsp11 Protein Mediates the Degradation of p21 Protein Dependent on Its Endoribonuclease Activity

**Figure 5:**
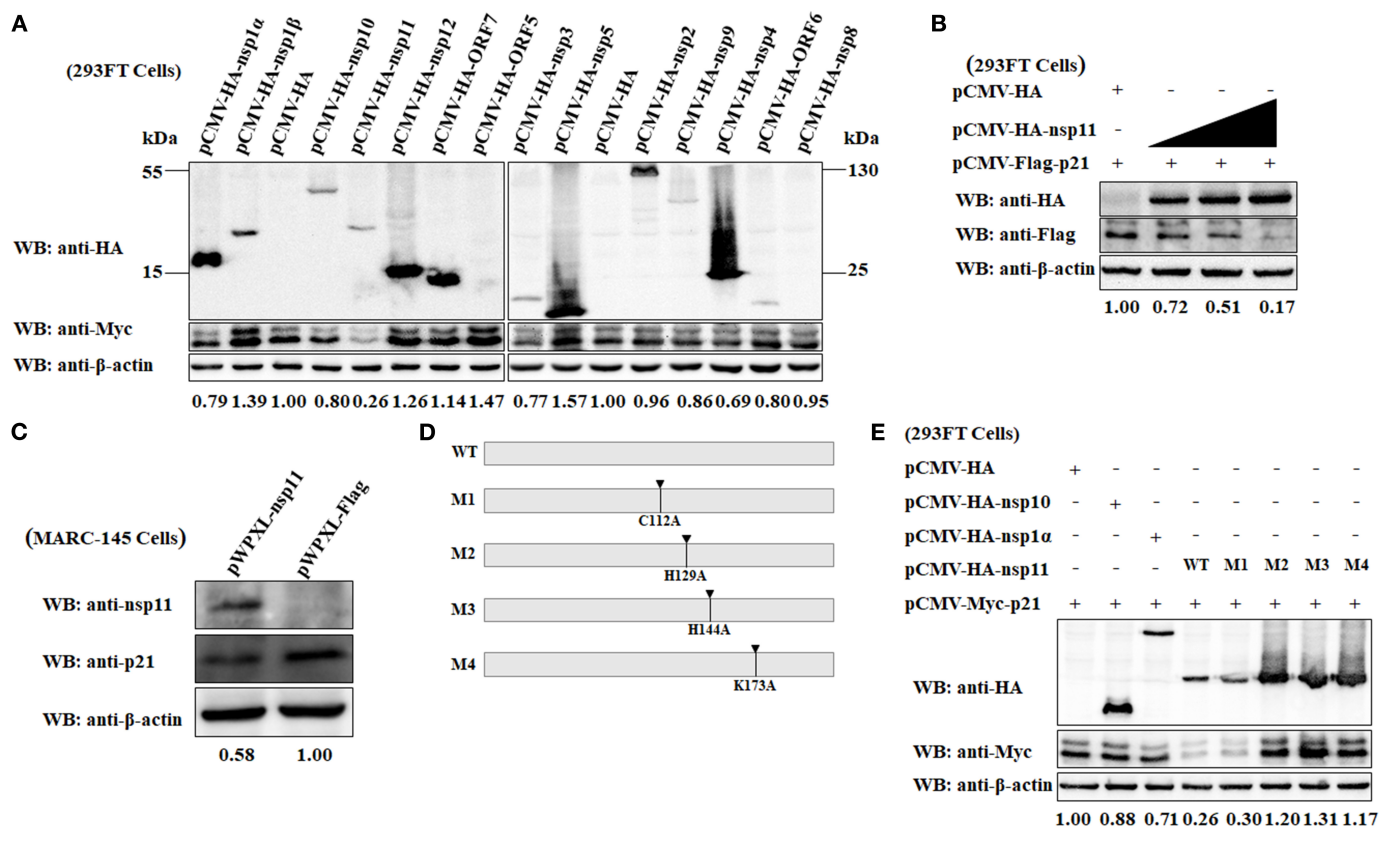
The PRRSV nsp11 protein mediates the degradation of p21 protein dependent on its endoribonuclease activity. **(A)** Identification of PRRSV protein that mediates the degradation of p21 protein. A series of recombinant pCMV-HA plasmids expressing HA-tagged each of individual major coding proteins of PRRSV were separately transfected into 293FT cells together with the Myc-tagged pCMV-Myc-p21 plasmid. At 24 hpt, the cells were harvested and subjected to western blot analysis using anti-HA, anti-Myc and anti-β-actin primary antibodies. β-Actin was used as a protein loading control. The densitometry ratios of p21/β-actin are indicated below the corresponding protein bands. **(B)** The PRRSV nsp11 protein mediates the degradation of p21 protein in a dose-dependent manner. 293FT cells grown in 6-well cell plates were co-transfected with 0.5 μg/well of pCMV-Flag-p21 plasmid and 0, 0.5, 1, or 2 μg/well of pCMV-HA-nsp11 plasmid. At 24 hpt, the cells were harvested and subjected to western blot analysis using anti-HA, anti-Flag and anti-β-actin primary antibodies. β-Actin was used as a protein loading control. The densitometry ratios of p21/β-actin are indicated below the corresponding protein bands. **(C)** MARC-145 cells constitutively expressing the PRRSV nsp11 protein exhibited a low expression level of endogenous p21 protein. The cell lysates of MARC-145 cells transduced with the recombinant lentiviruses expressing nsp11 (pWPXL-nsp11) or Flag (pWPXL-Flag) were subjected to western blot analysis using anti-nsp11, anti-p21, and anti-β-actin primary antibodies. **(D)** Construction strategy of the four nsp11 mutants (M1, M2, M3, and M4) with a single-site mutation of C112A, H129A, H144A, and K173A, respectively, using pCMV-HA-nsp11 as the backbone plasmid. **(E)** The PRRSV nsp11-mediated degradation of p21 protein is dependent on the endonuclease activity of nsp11 rather than its deubiquitinating activity. 293FT cells were co-transfected with pCMV-Myc-p21 and any one of the following plasmids pCMV-HA-nsp10, pCMV-HA-nsp1α, pCMV-HA-nsp11 or its four mutants (M1, M2, M3, and M4). At 24 hpt, the cells were lysed and subjected to western blot analysis using primary antibodies against HA, Myc and β-actin. β-Actin was used as a protein loading control. The densitometry ratios of p21/β-actin are indicated below the corresponding protein bands.

To elucidate which viral protein is responsible for the downregulation of p21 protein in MARC-145 cells, a series of eukaryotic plasmids expressing HA-tagged each of individual major coding proteins of PRRSV were separately transfected into 293FT cells together with the pCMV-Myc-p21 plasmid. As shown in Figure 5A, the expression level of Myc-tagged p21 protein in the MARC-145 cells co-transfected with pCMV-Myc-p21 and pCMV-HA-nsp11 was significantly lower than that of the control cells co-transfected with pCMV-Myc-p21 and empty plasmid pCMV-HA. In contrast, the cells co-transfected with pCMV-Myc-p21 and other plasmids expressing any other PRRSV protein other than nsp11 exhibited comparable expression levels of Myc-tagged p21 protein to the control cells. Moreover, we also evaluated the effect of different transfection doses of pCMV-HA-nsp11 on the expression level of p21 protein. Notably, to exclude the possibility of Myc tag affected the expression of p21 protein, we simultaneously constructed a Flag-tagged plasmid pCMV-Flag-p21, which was transfected into 293FT cells together with the pCMV-HA-nsp11 plasmid. The results showed that the higher the transfection dose of pCMV-HA-nsp11, the more obvious the degradation of p21 protein (Figure 5B), revealing that nsp11 protein degrades p21 protein in a dose-dependent manner. To further confirm our results obtained by overexpressing nsp11 and p21 proteins via a transfection manner, we proceeded to construct stable MARC-145 cells constitutively expressing the PRRSV nsp11 protein using a lentivirus system. Western blot analysis showed that the expression level of endogenous p21 protein in the constructed MARC-145 cells constitutively expressing nsp11 protein (pWPXL-nsp11) was significantly lower than that of the empty vector control cells (pWPXL-Flag; Figure 5C). This result suggests that the PRRSV nsp11 protein indeed mediates the downregulation of p21 protein in MARC-145 cells.

Because the PRRSV nsp11 protein possess endoribonuclease activity (4, 53), it is necessary to clarify whether nsp11-mediated degradation of p21 is dependent on its enzymatic activity. A previous study has demonstrated that there exist three key amino acid residues at positions 129 (His), 144 (His) and 173 (Lys) of the PRRSV nsp11 protein, which are closely related to the endoribonuclease activity of nsp11 (53). Replacement of any one of the three residues with alanine (Ala) was able to abrogate its endoribonuclease activity (54). Accordingly, we constructed three nsp11 mutants in which the endoribonuclease activity was abolished by introducing single-site mutations H129A, H144A, and K173A using pCMV-HA-nsp11 as the backbone plasmid (Figure 5D). The resulting mutant plasmids were designated M2, M3, and M4, respectively. In addition to the endoribonuclease activity, the PRRSV nsp11 protein has also been demonstrated to have deubiquitinating activity which could be inactivated by mutating a key amino acid residue at position 112 from Cys to Ala (55). To determine whether the deubiquitinating activity of nsp11 is also involved in nsp11-mediated degradation of p21 protein, we therefore constructed a mutant pCMV-HA-nsp11 plasmid with C112A mutation which was named as M1 (Figure 5D). The four mutants (M1, M2, M3, and M4) of plasmid pCMV-HA-nsp11 were separately transfected into 293FT cells together with the pCMV-Myc-p21 plasmid. As shown in Figure 5E, compared with the wild-type nsp11 protein (WT) produced by transfection with pCMV-HA-nsp11, the three mutants of nsp11 with impaired endoribonuclease activity, M2, M3, and M4, lost the ability to mediate the degradation of p21 protein. As expected, the cells that were co-transfected with the plasmid pCMV-HA-nsp10 or pCMV-HA-nsp1α (as controls of viral protein that did not mediate p21 degradation) or pCMV-HA (as an empty vector control) and pCMV-Myc-p21 did not display effect on p21 protein degradation. These results indicate that the PRRSV nsp11 protein mediated the degradation of p21 via its endoribonuclease activity. Moreover, it is noteworthy that p21 degradation still occurred in the cells transfected with the M1 mutant of pCMV-HA-nsp11 plasmid whose deubiquitinating activity was abolished. This reveals that the nsp11-mediated degradation of p21 protein is independent of the deubiquitinating activity.

### The PRRSV nsp11 Protein Mediates p21 Degradation *via* a Ubiquitin-Independent Proteasomal Degradation Manner

**Figure 6:**
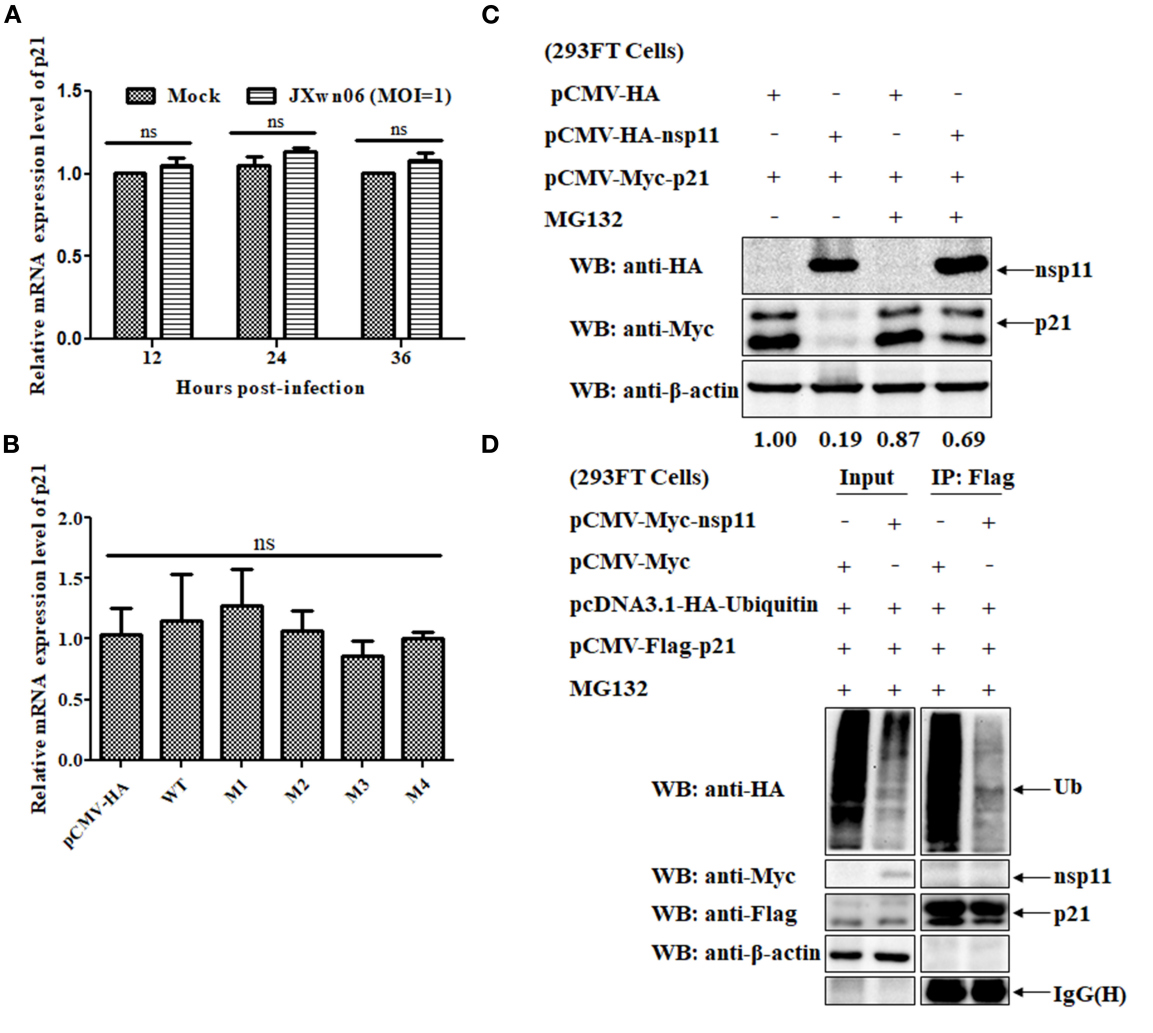
The PRRSV nsp11 protein mediates p21 degradation via a ubiquitin-independent proteasomal degradation manner. **(A)** Real-time PCR analysis of the relative mRNA expression level of p21 in mock- and PRRSV-infected MARC-145 cells. MARC-145 cells were mock infected or infected with the HP-PRRSV JXwn06 strain at an MOI of 1 TCID_50_/cell. At 12, 24, and 36 hpi, total cellular RNAs were extracted and reverse-transcribed into cDNA for real-time PCR analysis. Fold-change values were calculated based on the 2^−ΔΔCt^ method, using β-actin as the housekeeping gene. Error bars indicate the standard error of three independent experiments (two-way ANOVA test; ns, no significant difference). **(B)** Real-time PCR analysis of the relative mRNA expression level of p21 in the MARC-145 cells co-transfected with plasmids pCMV-Myc-p21 and pCMV-HA-nsp11 or its derivative mutants or pCMV-HA (empty vector control). **(C)** Western blot analysis of the effect of proteasomal pathway on the degradation of p21 protein. 293FT cells were co-transfected with pCMV-Myc-p21 and pCMV-HA-nsp11 or pCMV-HA (empty vector control) for 20 h, and then treated with 10 μM of MG132 or DMSO for 4 h. The cell lysates were determined by western blot analyses using anti-HA, anti-Myc and anti-β-actin primary antibodies. β-Actin was used as a protein loading control. The densitometry ratios of p21/β-actin are indicated below the corresponding protein bands. **(D)** 293FT cells grown in 6-well cell plates were co-transfected with 1 μg/well of pcDNA3.1-HA-Ubiquitin, 1 μg/well of pCMV-Flag-p21 and 1 μg/well of pCMV-Myc-nsp11. At 20 hpt, the cells were treated with 10 μM of MG132 for 4 h, and then harvested, immunoprecipitated with an anti-Flag mAb and protein A/G Sepharose beads. The immune complexes were separated on 12% SDS-PAGE gels and then analyzed by western blot analysis using primary antibodies against HA, Myc, Flag and β-actin.

To clarify whether the downregulation of p21 protein is related to an increased degradation of p21 protein or its mRNA, real-time PCR assays were performed to determine the relative mRNA expression level of p21 in mock- and PRRSV-infected MARC-145 cells. As shown in Figure 6A, there is no significant difference in the relative mRNA expression level of p21 between mock- and PRRSV-infected MARC-145 cells (*p* > 0.05). Moreover, we also detected the relative mRNA expression level of p21 in the MARC-145 cells co-transfected with plasmids pCMV-Myc-p21 and pCMV-HA-nsp11 or its derivative mutants, but no significant difference was found between different cell groups (Figure 6B). These results suggest that the downregulation of p21 protein is closely related to degradation of the protein rather than the mRNA.

Because the proteasome system is one of the most important pathways responsible for degradation of intracellular proteins in eukaryotic cells (54), it is of great significance to test whether the proteasomal pathway is involved in the nsp11-induced degradation of p21 protein. To this end, MG132, a well-known proteasome inhibitor, was used to treat the cells. Specifically, 293FT cells were co-transfected with pCMV-Myc-p21 and pCMV-HA-nsp11 or pCMV-HA, and then treated with 10 μM of MG132 or DMSO, the solvent control, for 4 h. The cell lysates were determined by western blot analyses. As shown in Figure 6C, MG132 treatment significantly recovered the expression level of Myc-tagged p21 protein in 293FT cells co-transfected with pCMV-Myc-p21 and pCMV-HA-nsp11 plasmids, indicating that the proteasomal pathway is involved in nsp11-mediated degradation of p21 protein. It should be noted that although the majority of proteins degraded via the proteasomal pathway need to be ubiquitinated before degradation (56), some proteins could be directly degraded by the proteasome without the need of prior modification by polyubiquitin (57). Accordingly, we proceeded to detect the ubiquitination status of p21 protein in the presence of nsp11 protein. 293FT cells were co-transfected with plasmids pcDNA3.1-HA-Ubiquitin, pCMV-Flag-p21 and pCMV-Myc-nsp11 or pCMV-Myc for 20 h and then treated with MG132 for 4 h. The cell lysates were used for Co-immunoprecipitation (Co-IP) assays. As shown in Figure 6D, the addition of MG132 successfully inhibited nsp11-mediated p21 degradation in the cells co-transfected with plasmids pcDNA3.1-HA-Ubiquitin, pCMV-Flag-p21 and pCMV-Myc-nsp11. Of note, the immune complexes pulled down by an anti-Flag mAb contained undetectable ubiquitinated p21 protein, indicating that the degradation of p21 protein by nsp11 has nothing to do with ubiquitination. Taken together, these results suggest that nsp11 mediates proteasomal degradation of p21 protein in a ubiquitin-independent manner.

## Discussion

Cell cycle refers to a series of events in which the genetic material of a cell is replicated and then equally divided into two daughter cells (25). After a continuous evolution, the eukaryotic cells have evolved a complex regulatory system to control the cell cycle progression (25). Because the virus cannot survive without the host cells, most viruses have evolved diverse strategies to modulate cellular machineries for their own replication (33–37). Although the interactions between PRRSV and its host cells are highly complex, investigating the effect of virus infection on host cells from the perspective of cell cycle regulation will provide valuable clues for further investigation of PRRSV pathogenesis.

As a well-known inhibitor of the cell cycle, p21 protein can cause cell cycle arrest at the G1 phase and thus block the cell cycle transition from G1 phase to S phase, and downregulation of p21 could antagonize this inhibition (52). In other words, the alteration of p21 protein expression can reflect the change of the cell cycle to some extent. In the present study, we first demonstrated that both the HP-PRRSV strain JXwn06 and the LP-PRRSV strain HB-1/3.9 are able to downregulate p21 protein in the permissive MARC-145 cells, which are widely used for studying PRRSV infection *in vitro*. To confirm whether the downregulation of p21 affected the cell cycle of MARC-145 cells, we designed p21-specific siRNAs to specifically silence p21 and found that knockdown of p21 promotes MARC-145 cells into S phase. Based on these findings, we speculate that PRRSV promotes MARC-145 cells entry into S phase of the cell cycle through degradation of p21. Next, we employed flow cytometry to analyze the effect of PRRSV infection on the cell cycle of MARC-145 cells, and discovered that PRRSV infection indeed promotes MARC-145 cells into S phase. These findings are consistent with a previous study indicating that the PRRSV nsp11 protein can induce cell-cycle arrest at S phase in MARC-145 cells constitutively expressing nsp11 (24). To date, a number of viruses, including high-risk human papillomavirus (HPV) (58–60), hepatitis B virus (61, 62), mouse hepatitis virus (63), influenza A virus (64), transmissible gastroenteritis virus (65), hepatitis C virus (HCV) (66–69), have been reported to have evolved various strategies to regulate the cell cycle by targeting CKIs. For example, the oncoprotein E7 of HPV was demonstrated to be able to abrogate p21-mediated inhibition of CDK2 and PCNA-dependent DNA replication via interaction with the C-terminus of p21, thereby promoting S-phase entry in human keratinocytes (58, 59). Moreover, the HPV-16 E7 oncoprotein also has a role in inducing S-phase entry by inactivating p27 via binding to the C-terminal region of E7 (60). Interestingly, our present study demonstrated that PRRSV infection has a slight effect on the expression of p27 in MARC-145 cells. Accordingly, we deduce that PRRSV promotes the entry of MARC-145 cells into S phase of the cell cycle mainly dependent on p21-mediated inhibition of CKIs. Notably, another study demonstrated that both CKIs and pRb are involved in regulation of the cell cycle in HCV-infected human hepatoma cells (69). The HCV RNA-dependent RNA polymerase, non-structural protein 5B (NS5B), was shown to interact with pRb through the LXCXE motif to induce E6AP-dependent degradation of pRb via the ubiquitin-proteasome pathway and thus promote hepatocellular proliferation (69). Similarly, one of our previous studies demonstrated that the PRRSV RNA-dependent RNA polymerase, non-structural protein 9 (nsp9), is also able to degrade pRb in PRRSV-infected MARC-145 cells in a way similar to the HCV NS5B (70). However, the effect of nsp9-mediated pRb degradation on the cell cycle warrants further investigation.

To elucidate the potential biological significance of S-phase entry on the replication of PRRSV in MARC-145 cells, we evaluated the ability of synchronized cells in different phases of the cell cycle to support PRRSV replication. Our results showed that MARC-145 cells in S phase exhibited the highest ability to support PRRSV replication among the synchronized cells in the G0/G1, S and G2/M phases of the cell cycle (Figure 4C). These results are consistent with those of two previous studies, in which the investigators demonstrated that enterovirus 71 and dengue virus type 2 (DEN2) are also able to induce S-phase entry in host cells to facilitate their own replication (71, 72). Interestingly, the S-phase-dependent enhancement of DEN2 replication was found to only occur in C6/36 mosquito cells, not in human hepatoma cells or primary human fibroblasts (72). These findings suggest that the roles played by the cell cycle, even in the same phase, in viral replication exhibit cell-type specific features. To further explore which viral protein is responsible for the downregulation of p21, we first constructed a series of eukaryotic plasmids expressing each of individual major coding proteins of PRRSV, and tested their ability to mediate p21 downregulation. Fortunately, we successfully discovered that the nsp11 protein of PRRSV is able to mediate p21 downregulation. On this basis, we focused our attention on elucidating how nsp11 functions to downregulate p21 protein expression in MARC-145 cells, thereby regulating the replication of PRRSV. Although we can't rule out the possibility that there might exist another protein out of the remaining PRRSV proteins, which haven't been evaluated in the present study, also being able to mediate the downregulation of p21 protein, the current experimental data clearly demonstrated that PRRSV promotes MARC-145 cells entry into S phase of the cell cycle to facilitate viral replication via degradation of p21 by nsp11.

Existing studies have shown that the PRRSV nsp11 protein has two well-known biological functions—RNA endoribonuclease activity and deubiquitinating activity (4, 53, 55). To determine which activity is involved in nsp11-mediated degradation of p21 in PRRSV-infected MARC-145 cells, we carried out mutational analyses of nsp11 through construction of endoribonuclease or deubiquitinating activity-inactivated nsp11 mutants, and discovered that nsp11-mediated p21 protein degradation is dependent on the endoribonuclease activity rather than the deubiquitinating activity. Since the degradation of any protein can occur at either the RNA or protein level or both, it is meaningful to clarify which way the nsp11-mediated degradation of p21 follows. To do this, we used western blot and real-time PCR assays to respectively, evaluate the changes of mRNA and protein expression levels of p21 in MARC-145 cells upon PRRSV infection or recombinant-plasmid transfection. Our results showed that the protein expression level of p21 rather than its mRNA expression level was evidently decreased when MARC-145 cells were exposed to PRRSV infection or recombinant-plasmid transfection (Figures 5B,E, 6A,B). These data suggest that the nsp11-mediated downregulation of p21 protein occurred at the protein level. Interestingly, a previous study discovered that the nsp11 protein of PRRSV is able to specifically degrade two innate immune molecules, mitochondrial anti-viral signaling protein (MAVS) and retinoic acid-inducible gene I (RIG-I), in both nsp11-gene transfected MARC-145 cells and PRRSV-infected cells (73). This study further demonstrated that the nsp11-mediated specific degradation of MAVS and RIG-I is dependent on the functional endoribonuclease activity of nsp11 protein, which first causes the degradation of MAVS and RIG-I mRNAs, thereby leading to the reduction of protein expressions (73). In contrast, our present study showed that although the endoribonuclease activity of nsp11 played an essential role for the downregulation of p21 protein (Figures 5D,E), it did not cause degradation of p21 mRNA (Figures 6A,B). Accordingly, we speculate that the PRRSV nsp11 protein mediates the degradation of p21 protein in a manner different from MAVS and RIG-I proteins. There might exist some intermediate molecules—whose mRNAs were degraded by the endoribonuclease activity of nsp11—that interact with nsp11 and thus jointly mediated p21 protein degradation in MARC-145 cells. Obviously, further investigation is needed to verify this speculation.

It is well-known that selective degradation of most intracellular proteins is implemented by the proteasome system (54), which comprises two distinct proteolytic pathways, one is the ubiquitin-dependent proteasomal degradation pathway (56), the other is the ubiquitin-independent proteasomal degradation pathway (57). For p21 protein, there is considerable evidence indicating that proteasomal degradation of p21 does not require the ubiquitylation of p21 protein (74–77); however, other reports suggest that proteasomal degradation of p21 protein requires its prior ubiquitination (78, 79). These facts reveal that the degradation of cellular p21 protein might be associated with cell types and/or stimuli. To find out which proteasome degradation pathway is involved in the PRRSV nsp11-mediated degradation of p21 protein, we evaluated the ubiquitinated forms of the degraded p21 protein mediated by nsp11 using Co-IP assays. We eventually demonstrated that nsp11 mediates the degradation of p21 protein through a ubiquitin-independent proteasomal degradation pathway.

It is important to note that it would be much more meaningful if our experimental data obtained with MARC-145 cells were further validated in primary PAMs, the natural host cells of PRRSV infection *in vivo*. Frankly speaking, we made an attempt to test the changes of p21 protein expression in PRRSV-infected PAMs using western blot analyses (data not shown), and the results were similar to those obtained with MARC-145 cells. However, the other important experimental results were not successfully obtained with the primary PAMs, because PAMs are too fragile to withstand the relevant experiments such as RNA interference, transfection and cell cycle synchronization. In view of this, we chose to use the permissive cells of PRRSV, MARC-145, which are widely for the basic research and *in vitro* cultivation of PRRSV, to investigate the interactions between PRRSV infection and cell-cycle phases.

In summary, the present study demonstrated that PRRSV infection can promote MARC-145 cells entry into S phase of the cell cycle, which are conducive to the replication of PRRSV. The promotion of MARC-145 cells entry into the S phase was caused by the degradation of p21 by nsp11. The nsp11-mediated degradation of p21 was in an ubiquitin-independent and proteasome-dependent degradation manner, which requires the endoribonuclease activity of nsp11. Undoubtedly, our study provides a better understanding of the interactions between PRRSV and its host cells.

## Data Availability Statement

The original contributions presented in the study are included in the article/Supplementary Material, further inquiries can be directed to the corresponding author/s.

## Author Contributions

XW and XGe performed experiments. XW, XGe, LZ, YZ, and XGu analyzed data. XW and YZ wrote the paper. HY designed research and edited the paper. All authors have read and approved the final manuscript.

## Conflict of Interest

The authors declare that the research was conducted in the absence of any commercial or financial relationships that could be construed as a potential conflict of interest.

## Supplementary Material

Supplementary Figure 1 Analysis of the silencing efficiency of p21 protein using different doses of siRNAs. MARC-145 cells were transfected with 20, 30, or 40 pmol of each siRNA (Si-215, Si-462, and Si-432) or scrambled siRNA (Si-NC). At 36 hpt, the cells were harvested and processed for western blot analyses using an anti-p21 pAb. β-Actin was used as a protein loading control. The densitometry ratios of p21/β-actin are indicated below the corresponding protein bands.

Supplementary Figure 2 Construction of p21 gene-knockout MARC-145 cells using CRISPR/Cas9 technology. **(A)** Schematic illustration of hCas9 (D10A) inactivation of the p21-gene locus. The shown sequence is part of the exon 1 of p21 gene. The numbers indicate the nucleotide positions in the p21-gene open reading frame. The 20-bp sgRNA target sequences are shown in green, and the protospacer-adjacent motifs (PAM) are shown in red. The red arrows indicate the putative cleavage sites. **(B)** DNA sequencing of the p21-gene locus surrounding the sgRNA sites of the constructed p21-knockout MARC-145 cells. The genomic fragment encompassing the CRISPR target sequence was PCR-amplified from the genomic DNA of the constructed p21-knockout MARC-145 cells. The inserted bases are shown with a red frame.
