# Supplementary figures and images for: PRRSV Promotes MARC-145 Cells Entry Into S Phase of the Cell Cycle to Facilitate Viral Replication via Degradation of p21 by nsp11
Source: Front Vet Sci. 2021 Mar 24;8:642095. doi: 10.3389/fvets.2021.642095 (PMC8044838; doi:10.3389/fvets.2021.642095)

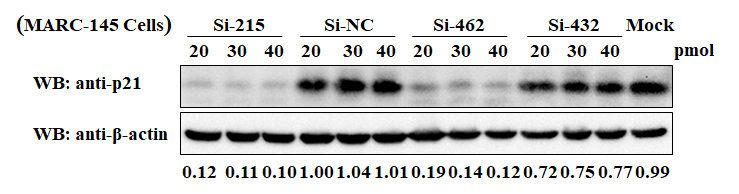

Supplement: Supplementary Figure 1 — Analysis of the silencing efficiency of p21 protein using different doses of siRNAs. MARC-145 cells were transfected with 20, 30, or 40 pmol of each siRNA (Si-215, Si-462, and Si-432) or scrambled siRNA (Si-NC). At 36 hpt, the cells were harvested and processed for western blot analyses using an anti-p21 pAb. β-Actin was used as a protein loading control. The densitometry ratios of p21/β-actin are indicated below the corresponding protein bands. [file Image_1.TIF]

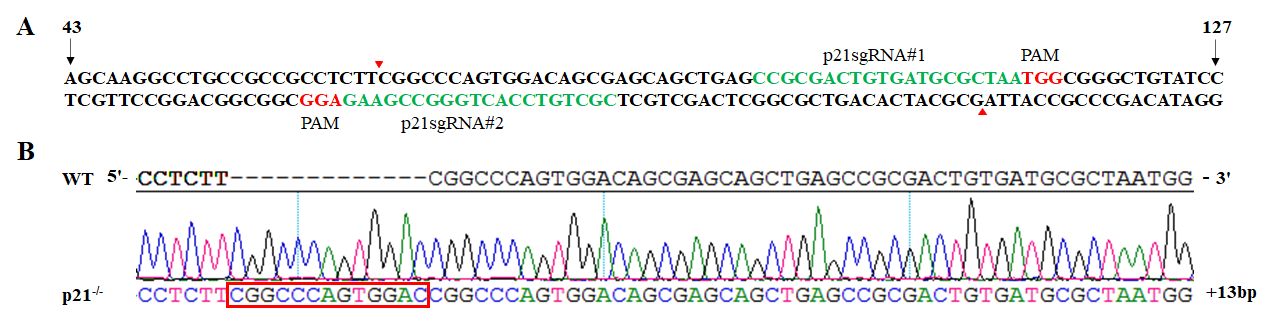

Supplement: Supplementary Figure 2 — Construction of p21 gene-knockout MARC-145 cells using CRISPR/Cas9 technology. (A) Schematic illustration of hCas9 (D10A) inactivation of the p21-gene locus. The shown sequence is part of the exon 1 of p21 gene. The numbers indicate the nucleotide positions in the p21-gene open reading frame. The 20-bp sgRNA target sequences are shown in green, and the protospacer-adjacent motifs (PAM) are shown in red. The red arrows indicate the putative cleavage sites. (B) DNA sequencing of the p21-gene locus surrounding the sgRNA sites of the constructed p21-knockout MARC-145 cells. The genomic fragment encompassing the CRISPR target sequence was PCR-amplified from the genomic DNA of the constructed p21-knockout MARC-145 cells. The inserted bases are shown with a red frame. [file Image_2.TIF]
